# The proteasome regulates body weight and systemic nutrient metabolism during fasting

**DOI:** 10.1152/ajpendo.00069.2023

**Published:** 2023-09-06

**Authors:** Henning Tim Langer, Samuel R. Taylor, Mujmmail Ahmed, Tiffany Perrier, Tanvir Ahmed, Marcus D. Goncalves

**Affiliations:** Department of Medicine, Weill Cornell Medicine, New York, New York, United States

**Keywords:** autophagy, fasting, glucose, proteasome, proteolysis

## Abstract

The ubiquitin-proteasome system (UPS) and the autophagy-lysosome pathway are the primary means of degradation in mammalian tissues. We sought to determine the individual contribution of the UPS and autophagy to tissue catabolism during fasting. Mice were overnight fasted for 15 h before regaining food access (“Fed” group, *n* = 6) or continuing to fast (“Fast” group, *n* = 7) for 3 h. In addition, to investigate the effects of autophagy on systemic metabolism and tissue degradation, one group of mice was fasted for 18 h and treated with chloroquine (“Fast + CLQ” group, *n* = 7) and a fourth group of mice was treated with bortezomib (“Fast + Bort” group, *n* = 7) to assess the contribution of the UPS. Body weight, tissue weight, circulating hormones and metabolites, intracellular signaling pathways, and protein synthesis were investigated. Fasting induced the loss of body weight, liver mass, and white adipose tissue in the Fast and the Fast + CLQ group, whereas the Fast + Bort group maintained tissue and body weight. Fasting reduced glucose and increased β hydroxybutyrate in the circulation of all mice. Both changes were most profound in the Fast + Bort group compared with the other fasting conditions. Molecular signaling indicated a successful inhibition of hepatic UPS with bortezomib and an upregulation of the PI3K/AKT/mTOR pathway. The latter was further supported by an increase in hepatic protein synthesis with bortezomib. Inhibition of the UPS through bortezomib blocks body weight loss and tissue catabolism during an acute overnight fast in mice. The effects were likely mediated through a combined effect of the drug on biomolecule degradation and synthesis.

**NEW & NOTEWORTHY** Bortezomib treatment prevents tissue and body weight loss during fasting. The loss of proteasome activity with bortezomib exacerbates fasting-induced ketogenesis. During fasting, bortezomib increases AMPK and PI3K/AKT signaling in the liver, which promotes protein synthesis.

## Graphical abstract

**Figure:**
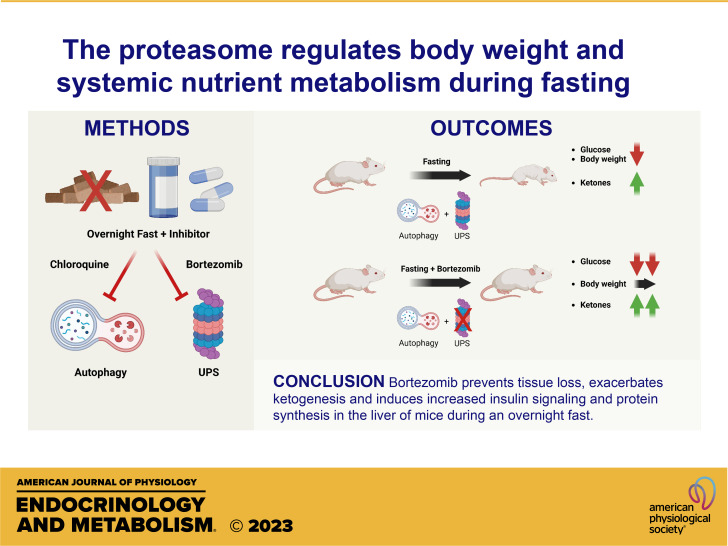

## INTRODUCTION

Food restriction activates a systemic, neurohormonal program that preserves the blood levels of essential nutrients like glucose. During times of starvation or fasting, changes in systemic hormones like insulin, catecholamines, and glucocorticoids reduce anabolism and activate catabolic pathways in tissues that serve as the body’s nutrient reservoirs. For example, low insulin and high glucocorticoid levels suppress anabolic and enhance catabolic processes in peripheral tissues resulting in degradation of proteins and release of amino acids into the blood. These amino acids can be used as substrates for gluconeogenesis by the liver and kidney, which sustains blood glucose when dietary nutrients are restricted.

Great efforts have been made to identify the molecular regulators of tissue catabolism, and two primary mechanisms have emerged: macroautophagy (furthermore referred to as autophagy) and the ubiquitin-proteasome system (UPS). Degradation through autophagy requires the formation of autophagosomes, which eventually fuse to lysosomes that contain hydrolytic enzymes (1). This process degrades glycogen granules, lipid droplets, and proteins to provide recycled glucose, free fatty acids, and amino acids for use in energy production and synthesis of new macromolecules. Although lysosomes degrade nearly all macromolecules, the UPS is the major pathway that facilitates the catabolism of ∼75% of intracellular proteins in eukaryotic cells (2, 3). During this process, E3 ubiquitin ligases covalently tag proteins to facilitate their delivery to the 26S proteasome (4, 5). Proteasome-catalyzed degradation of ubiquitin-conjugated proteins controls the size of cells by removing large proteins and smaller, short-lived proteins including transcription factors, proteins that govern the cell cycle and division, and those that regulate apoptosis (6).

As expected, both autophagy and UPS pathways are highly regulated in response to food intake (7). Following a meal, the incoming nutrients and subsequent activation of insulin and mTORC1 signaling results in decreased activity of UPS and autophagy (8–10). On the other hand, nutrient deprivation during periods of fasting initiates a complex signaling cascade through the central hub, AMPK, which increases the activity of the UPS and autophagy in a time- and dose-dependent manner (11, 12).

Altering the UPS and autophagy has been suggested as a potential strategies to treat disease states associated with tissue wasting. For example, the inhibition of the UPS with bortezomib can prevent tissue wasting as a consequence of denervation (13), muscular dystrophy (14, 15), burn injury (16), or sepsis (17) in animal models. Furthermore, bortezomib is approved as an anticancer treatment for multiple myeloma and mantle cell lymphoma (18, 19). Also, studies have convincingly shown that autophagy is required for the maintenance of systemic metabolism and tissue integrity during fasting and cancer, which both promote tissue wasting (20). Autophagy can be inhibited by chloroquine, a quinoline that accumulates within lysosomes as a deprotonated weak base and, thereby, increases lysosomal pH and impairs the fusion between autophagosome and lysosome (21).

Since both the UPS and autophagy are potently induced by nutrient deprivation and both lead to protein breakdown and tissue loss (22–28), it is unclear which of these two processes is more pronounced in modulating tissue wasting during acute starvation. To answer this question, we pharmacologically inhibited these pathways in fasted mice and investigated the resulting changes in body weight, tissue mass, tissue composition, serum metabolites, and molecular signaling pathways.

## METHODS

### Animal Work

Mice were maintained in temperature- and humidity-controlled specific-pathogen-free conditions on a 12-h light-dark cycle and received rodent chow (PicoLab Rodent 20 5053 LabDiet) and free access to drinking water. All animal studies were approved by the Institutional Animal Care and Use Committee (IACUC) of Weill Cornell Medical College and maintained as approved by the Institutional Animal Care and Use Committee (IACUC) at Weill Cornell Medicine under protocol number 2013-0116.

### Drug Administration

Chloroquine (VWR, TCC2301) and bortezomib (Fisher Scientific, NC9951119) were dissolved in sterile PBS and were administered via intraperitoneal injection at 50 and 1 mg/kg, respectively. Drug doses were chosen based on previous studies (29, 30). One dose was given at the initiation of the fast and another given 2 h before euthanasia, for a total of two doses. Sterile PBS was administered to mice not receiving drug.

### Fasting Intervention and Collection Protocol

**Figure 1.**
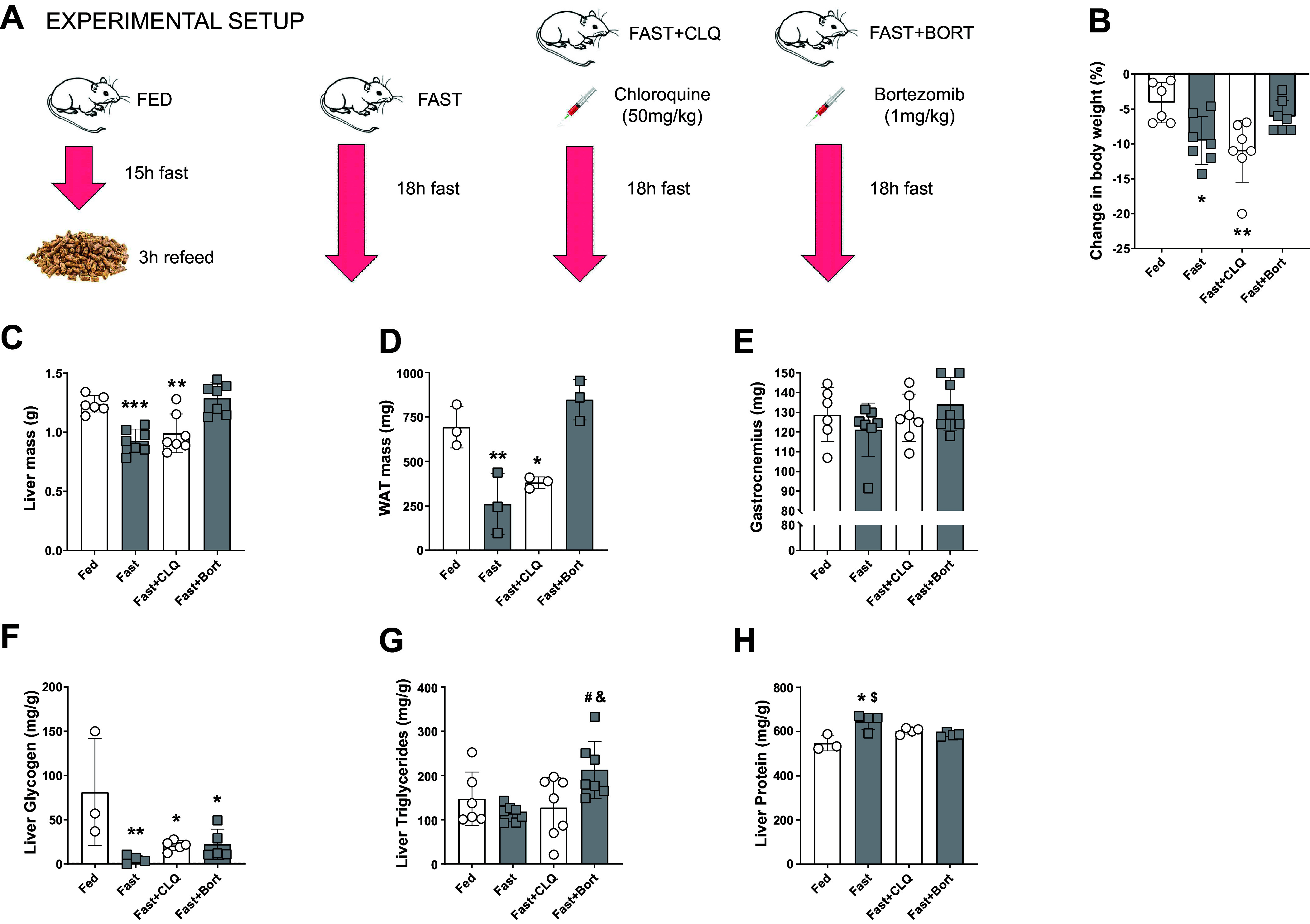
Bortezomib maintains tissue and body weight during an 18 h overnight fast. Experimental setup (*A*) and changes in body weight from the day before the start of the intervention (*B*). *B*: liver mass at the time of collection. Liver mass (*C*), white adipose tissue (WAT) mass (*D*), and gastrocnemius mass (*E*). Liver glycogen (*F*), triglyceride (*G*), and protein content (*H*). A one-way ANOVA and a Tukey post hoc multiple comparison test were used to assess group differences. Statistically significant differences to the Fed condition are denoted as *, **, and *** (*P* < 0.05, 0.01, or 0.001, respectively). Significant differences to the Fast group (*P* < 0.05) are denoted as #. Significant differences to the Fast+CLQ group (*P* < 0.05) are denoted as &. Significant differences to the Fast+Bort group (*P* < 0.05) are denoted as $. Bort, bortezomib; CLQ, chloroquine.

Male mice were subject to an overnight fast followed by a refeed period (FED; *n* = 6), an overnight fast without refeed (FAST; *n* = 7), an overnight fast plus chloroquine treatment (FAST + CLQ; *n* = 7), and overnight fast in addition to bortezomib treatment (FAST + BORT; *n* = 7). All animals were fasted for 15 h overnight on a wire grid. After the 15 h overnight fast, the FED group regained ad libitum food access for another 3 h before they were euthanized via CO_2_ and the tissue collected. All FAST groups were continuously fasted for a total duration of 18 h (Fig. 1*A*).

### Tissue Collection

Tail glucose was measured using a glucose meter (Ascensia) before CO_2_ asphyxiation. Immediately following euthanasia, whole blood was collected via cardiac puncture and placed immediately on ice. Next, the whole liver was removed, weighed, and frozen in liquid nitrogen (in under 30 s). The gonadal adipose depot, kidney, and skeletal muscles were dissected, weighed, and flash-frozen in liquid nitrogen. All tissues were subsequently stored at −80°C until further processing.

### Serum and Tissue Analysis

Blood was centrifuged (10,000 *g* for 10 min at 4°C), and plasma was stored at −20°C. Serum β hydroxybutyrate (BHB; Stanbio Laboratory), nonesterified fatty acids (NEFA; Wako Life Sciences), and lactate (Sigma-Aldrich) were determined using commercially available kits. Serum insulin (Crystal Chem, 90080) was quantified by ELISA. Tissue and serum triglycerides (TG; Stanbio Laboratory) and protein (Thermo Scientific) were quantified by commercial kit.

For glycogen measurements, liver tissue (30–50 mg) and dilutions of glycogen type III obtained from rabbit liver (Sigma-Aldrich) were homogenized in 0.03 N HCl. An aliquot of the homogenate was mixed with 1.25 N HCl and heated for 1 h at 95°C. Samples were centrifuged at 18,400 *g*, and 10 µL of supernatant was mixed with 1 mL of glucose oxidase reagent (Stanbio Laboratory). After a short incubation at 37°C, the absorbance was read at 505 nm.

### Metabolomics

Polar metabolite profiling was performed according to a method described in a previous publication (31). Metabolites were extracted from serum using prechilled 80% methanol (−80°C). The extract was dried completely with a Speedvac at room temperature. The dried sample was redissolved in HPLC grade water before it was applied to the hydrophilic interaction chromatography LC-MS. Metabolites were measured on a Q Exactive Orbitrap mass spectrometer (Thermo Scientific), which was coupled to a Vanquish UPLC system (Thermo Scientific) via an Ion Max ion source with a HESI II probe (Thermo Scientific). A Sequant ZIC-pHILIC column (2.1 mm i.d. × 150 mm, particle size of 5 µm, Millipore Sigma) was used for separation of metabolites. A 2.1 × 20 mm guard column with the same packing material was used for protection of the analytical column. Flow rate was set at 150 μL/min. Buffers consisted of 100% acetonitrile for mobile phase A and 0.1% NH_4_OH/20 mM CH_3_COONH_4_ in water for mobile phase B. The chromatographic gradient ran from 85% to 30% A in 20 min followed by a wash with 30% A and reequilibration at 85% A. The Q Exactive was operated in full scan, polarity-switching mode with the following parameters: the spray voltage 3.0 kV, the heated capillary temperature 300°C, the HESI probe temperature 350°C, the sheath gas flow 40 units, the auxiliary gas flow 15 units. MS data acquisition was performed in the mass-to-charge ratio (*m*/*z*) range of 70–1,000, with 70,000 resolution (at 200 *m*/*z*). The automatic gain control (AGC) target was 1e6, and the maximum injection time was 250 ms. The MS data were processed using XCalibur 4.1 (Thermo Scientific) to obtain the metabolite signal intensity for relative quantitation. Metabolites were identified using an in-house library established using chemical standards. Identification required exact mass (within 5 ppm) and standard retention times.

### Western Blot

Liver and muscle tissue were lysed using lysis buffer containing 50 mM Tris·HCl (pH 7.4), 150 mM NaCl, 1 mM EDTA, 10% glycerol, 1% NP-40, 0.5% Triton X-100, and 1 tablet (per 10 mL) of protease and phosphatase inhibitor. Protein extracts (50 μg) were separated by 4%–12% NuPAGE Bis-Tris gel (Invitrogen, Carlsbad, CA) and transferred to 0.45-μm PVDF membranes with wet transfer cells (Bio-Rad Laboratories, Hercules, CA). After 1 h of blocking with Tris-buffered saline with 0.1% (vol/vol) Tween 20 (TBST) containing 5% (wt/vol) BSA, membranes were incubated overnight at 4°C with primary antibodies at a 1:1,000 dilution in 5% BSA followed by a TBST wash and the appropriate secondary antibody (1:3,000) for 1 h at room temperature. The signals were detected on HyBlot CL Autoradiography Film (Denville Scientific Holliston, MA) with SuperSignal Western Blot enhancer solution (Thermo Fisher, Waltham, MA).

### Protein Synthesis

Global liver protein synthesis was assessed using the SUrface SEnsing of Translation (SUnSET) method as described previously (32, 33). Puromycin was dissolved in sterile saline (0.9% NaCl) and delivered via intraperitoneal injection (0.02 μmol puromycin per g body weight) 30 min before tissue collection. Puromycin-truncated peptides, reflecting the rate of global tissue protein synthesis, were analyzed by Western blot as described earlier.

### Antibodies

The following antibodies were used in this study: 4EBP1, CST 9452; ACC, CST 3662; AKT, CST 9272; Ampka2, CST 2757; AS160, CST 2670; LC3B, CST 3868; P-4EBP1 (T37/46), CST 2855; p62, CST 5114; P-ACC (S79), CST 11818; P-AKT (S473), CST 4058; P-AMPKa (T172), CST 2535; P-PRAS40 (T246), CST 13175; PRAS40, CST 2691; Ubiquitin, CST 3933; puromycin, Millipore Sigma MABE343; P-AS160 (T642), Sigma 07-802.

### Statistics

All summary data are expressed as individual data points and means ± SD. Group means were compared through a one-way ANOVA for main effects in combination with Tukey’s post hoc test to investigate differences between individual groups. Prism 9 (GraphPad La Jolla, CA) was used to perform statistical analysis. The threshold for statistical significance was set to *P* < 0.05 and is indicated in figures using asterisks.

## RESULTS

### Blocking the Proteasome but Not Autophagy Exacerbates the Metabolic Response to Fasting

To activate autophagy and the UPS, mice were fasted for 18 h overnight (Fast; Fig. 1*A*). The mice were compared with a group that was fasted for 15 h and then regained food access for 3 h before collection (Fed). Fed mice served as a negative control in our experiment because autophagy and the UPS flux should be low in that state (34). To establish the individual contribution of autophagy and the UPS to the metabolic changes occurring during fasting and to see whether we could prevent tissue wasting by blocking one or the other, we treated two additional groups of fasted mice with either chloroquine (Fast + CLQ) or bortezomib (Fast + Bort).

We first analyzed the effects of our interventions on circulating nutrients and hormones (Table 1). Since circulating glucose is a primary source of energy for tissues and commonly decreases robustly with starvation (35), we looked at changes in tail vein glucose between the onset of the intervention and the point of collection. Glucose levels did not change significantly in the Fed mice (+7 ± 25%) but did dramatically drop in the Fast (−45 ± 25%), the Fast + CLQ (−47 ± 13%), and the Fast + Bort (−56 ± 21%) groups, as expected. The absence of ingested nutrients and the decrease in blood glucose during fasting commonly results in a concomitant decrease of circulating insulin (36). We found insulin levels to be lower in Fast (0.37 ± 0.19 ng/mL) and Fast + CLQ (0.36 ± 0.05 ng/mL) groups as compared with Fed mice (1.01 ± 0.27 ng/mL), however unchanged in the Fast + Bort (1.01 ± 0.24 ng/mL). These differences did not reach statistical significance. We measured lactate as a surrogate for systemic glucose metabolism (37) and found that it too was reduced in the fasted mice from 5.5 ± 0.1 µM in the Fed group to 2.4 ± 0.5 µM in the Fast, 2.5 ± 0.7 µM in the Fast + CLQ group, and trending lower to 1.9 ± 0.6 µM in the Fast + Bort group (38). These data are consistent with fasting causing a depletion of circulating glycolytic intermediates and TCA substrates, which is possibly accentuated by bortezomib.

**Table 1.** Overnight fasting induces robust hypoglycemia and hyperketonemia in mice, which is exacerbated by bortezomib but not chloroquine

	Fed	Fast	Fast + CLQ	Fast + Bort	*P* Value (ANOVA)
Glucose	7 ± 25%	−45 ± 15%***	−47 ± 13%***	−56 ± 21%****	<0.0001
Insulin	1.01 ± 0.27 ng/mL	0.37 ± 0.19 ng/mL	0.36 ± 0.05 ng/mL	1.01 ± 0.24 ng/mL	0.08
BHB	0.15 ± 0.02 mM	1.37 ± 0.29 mM*	1.37 ± 0.29 mM	1.37 ± 0.29 mM ***,##,&&	<0.001
NEFA	0.68 ± 0.08 mEq/L	1.32 ± 0.46 mEq/L	2.1 ± 1.09 mEq/L**	1.7 ± 0.52 mEq/L*	<0.01
TG	130 ± 26 mg/dL	115 ± 15 mg/dL	133 ± 20 mg/dL	147 ± 39 mg/dL	0.21
Lactate	5.5 ± 0.1 µM	2.4 ± 0.5 µM****	2.5 ± 0.7 µM****	1.9 ± 0.6 µM****	<0.001

Values are means ± SD. Changes in serum glucose from beginning till end of the experiment. Serum insulin, β hydroxybutyrate (BHB), nonesterified fatty acids (NEFA), triglycerides, and lactate levels at the point of tissue collection. Sample size was *n* = 6 or 7 animals/group. A one-way ANOVA and a Tukey post hoc multiple comparison test were used to assess group differences. Statistically significant differences to the Fed condition are denoted as *, **, ***, and **** (*P* < 0.05, 0.01, 0.001, or 0.0001, respectively). Significant differences to the Fast group are denoted as ## (*P* < 0.01). Significant differences to the Fast+CLQ group are denoted as && (*P* < 0.01). Bort, bortezomib; CLQ, chloroquine; TG, triglycerides.

In contrast to the decrease in glucose and insulin during fasting, hepatic fatty acid oxidation and ketone body production and release in the circulation increase during fasting (39, 40). The hydrolysis of triglycerides (TG) within adipocytes produces nonesterified fatty acids (NEFA), which are the primary substrates for hepatic fatty acid oxidation and ketone production (41–43). In our experiment, NEFA levels were high in all fasted mice, reaching statistical significance in the Fast + CLQ (2.1 ± 1.09 mEq/L) and Fast + Bort (1.7 ± 0.52 mEq/L) but not the Fast (1.32 ± 0.46 mEq/L) control as compared with the Fed group (0.68 ± 0.08 mEq/L). In keeping with previous reports (44, 45), 18 h of fasting was insufficient to elicit changes in serum TG levels. We found β hydroxybutyrate (BHB) to be high in all fasted groups, reaching statistical significance in Fast mice (1.37 ± 0.29 mM) and Fast + Bort (2.84 ± 0.87 mM), but not Fast + CLQ (1.09 ± 0.37 mM). Interestingly, the values of BHB in the Fast + Bort group were significantly higher than the other fasted groups suggesting a more severe carbohydrate depletion in those animals. Overall, these results indicate that fasting-induced fatty acid mobilization and metabolism are not impaired with chloroquine and appear to be exacerbated with bortezomib.

### Blocking the Proteasome but Not Autophagy Results in Preservation of Body Weight, Liver and Adipose Tissue Mass during Acute Fasting

Body weight significantly decreased in the Fast (−5%, *P* < 0.05) and Fast + CLQ (−7%, *P* < 0.01) groups as compared with the Fed condition (Fig. 1*B*). However, bortezomib preserved body weight during fasting (*P* = 0.71; Fig. 1*B*). Since liver, white adipose tissue (WAT), and skeletal muscle are major energy depots in mammalian bodies, we sought to reconcile the body weight changes on the individual tissue level. Liver mass was significantly reduced in the Fast (−31%, *P* < 0.001) and the Fast + CLQ groups (−25%, *P* < 0.01) but, like body weight, bortezomib completely preserved liver mass during fasting (+5%, *P* = 0.86; Fig. 1*C*). Similarly, fasting depleted WAT by more than half (*P* < 0.01) in the Fast and Fast + CLQ groups (*P* = 0.05), but remained unchanged with Fast + Bort (*P* = 0.44), as compared with Fed mice (Fig. 1*D*). Interestingly, the fasting intervention did not significantly alter gastrocnemius mass in any of the fasting groups (Fig. 1*E*). These data suggest that the proteasome regulates total body mass during fasting by mediating access to nutrient-rich tissues like the liver and adipose.

We next interrogated the macromolecule composition of the liver from mice in each intervention group. Fasting severely and significantly reduced liver glycogen levels in all three groups as compared with the Fed group (*P* < 0.05, Fig. 1*F*). To investigate whether blocking autophagy or UPS had an impact on hepatic lipid content, we measured intrahepatic TG levels. We found no change between Fed and the Fast and Fast + CLQ groups but detected significantly higher levels in Fast + Bort compared with the other two fasted groups (Fig. 1*G*) suggesting that bortezomib either impairs TG liberation into the circulation or hepatic TG oxidation (46). Finally, we measured protein concentrations to determine whether there was a preference for hepatic macromolecule preservation during fasting with or without inhibition of autophagy and UPS activity. We found that protein concentrations were highest in the fasting only group (645.9 mg/g, *P* < 0.01), followed by the Fast + CLQ group (602.2 mg/g, *P* < 0.05) whereas content was unchanged in the Fast + Bort group (586.4 mg/g, *P* = 0.17), as compared with the Fed group (547.8 mg/g, Fig. 1*H*). This relative rise in protein content of the fasted liver is likely a reflection of the severe glycogen depletion and overall loss in mass as compared with the other groups.

### Bortezomib Preferentially Reduces Circulating Metabolites of Glycolysis and Amino Acid Metabolism, Whereas It Increases the Abundance of Metabolites Associated with Fat Oxidation

**Figure 2.**
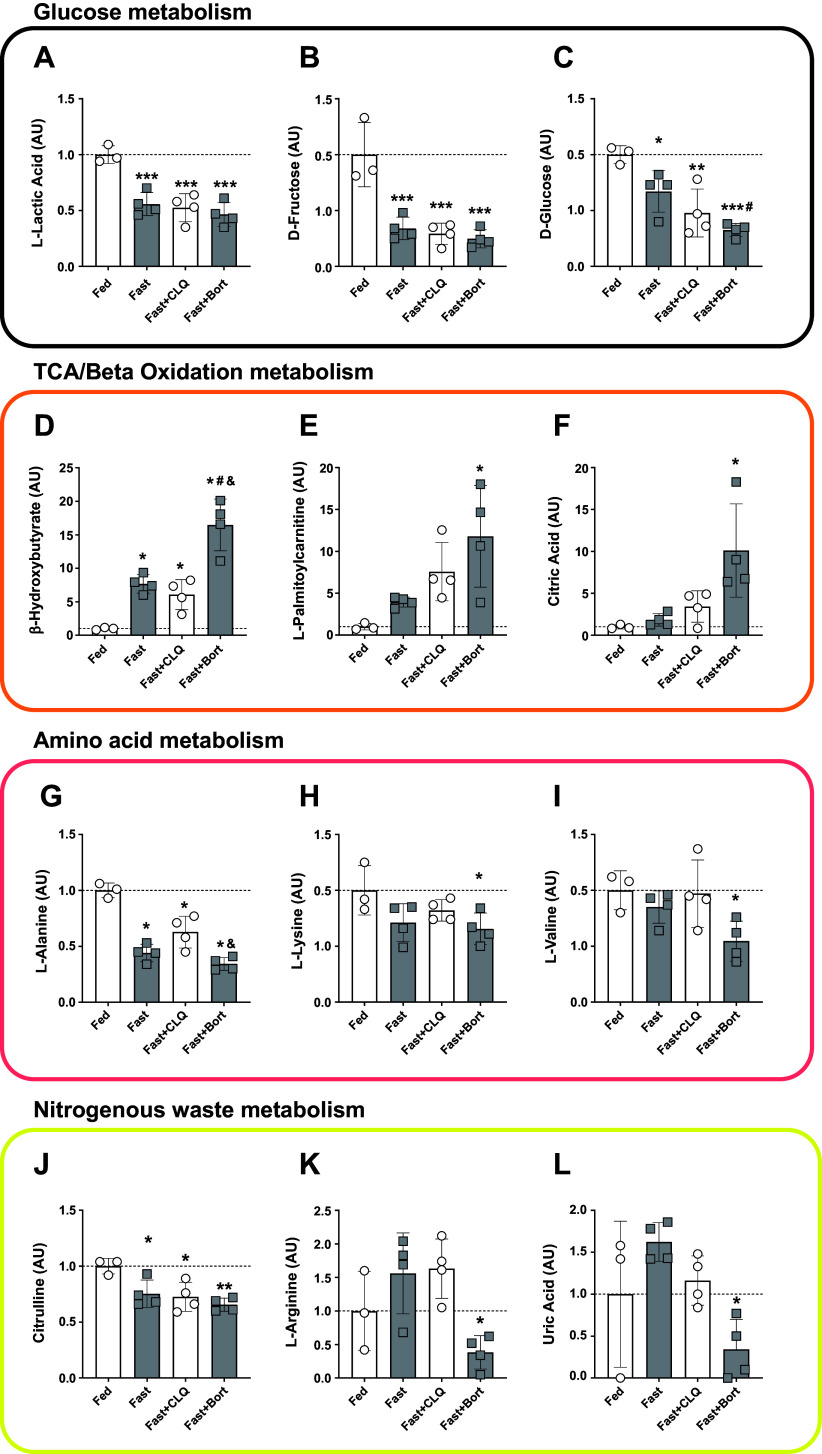
Bortezomib reduces circulating metabolites associated with glycolysis and proteolysis, while increasing the abundance of metabolites associated with fatty acid metabolism. Serum levels of metabolites associated with carbohydrate metabolism: lactic acid (*A*), fructose (*B*), and glucose (*C*). Serum levels of metabolites associated with oxidative metabolism: β hydroxybutyrate (*D*), palmitoylcarnitine (*E*), and citric acid (*F*). Serum levels of metabolites associated with amino acid metabolism: alanine (*G*), lysine (*H*), and valine (*I*). Serum levels of metabolites associated with the removal of nitrogenous waste: citrulline (*J*), arginine (*K*), and uric acid (*L*). A one-way ANOVA and a Tukey post hoc multiple comparison test were used to assess group differences. Statistically significant differences to the Fed condition are denoted as *, **, and *** (*P* < 0.05, 0.01, or 0.001, respectively). Significant differences to the Fast group (*P* < 0.05) are denoted as #. Significant differences to the Fast+CLQ group (*P* < 0.05) are denoted as &. Bort, bortezomib; CLQ, chloroquine.

We performed an unbiased metabolomics screen of the serum via LC-MS to investigate the abundance of circulating metabolites more thoroughly in our fasting experiment. We used a panel of targeted metabolites encompassing the key members of central carbon metabolism. In accordance with our analyses in Table 1, glycolytic metabolites were significantly decreased in all fasting groups (Fig. 2, *A*–*C*). The reduction in glucose was most pronounced in the Fast + Bort group (68% lower as compared with Fed, *P* < 0.001) and significantly stronger than the fasting alone group (*P* < 0.05; Fig. 2*C*). There was a significantly larger increase in circulating β hydroxybutyrate levels (Fig. 2*D*), which suggests a switch in hepatic metabolism toward fatty acid oxidation: although fasting alone elevated BHB to levels 7.7-fold higher than Fed mice (*P* < 0.05) and Fast + CLQ to sixfold higher (*P* < 0.05), bortezomib increased BHB to 17-fold higher levels (*P* < 0.0001). This robust increase inversely mirrors the decrease in glucose and made circulating BHB levels significantly higher in the Fast + Bort compared with fasting alone (*P* < 0.01) and fasting with chloroquine (*P* < 0.001; Fig. 2*D*). Similarly, metabolites from the TCA cycle (citric acid) and β oxidation (l-palmitoylcarnitine) were significantly increased with Fast + Bort but none of the other fasting conditions (Fig. 2, *E* and *F*).

In addition to carbohydrates and fatty acids, several amino acids were perturbed during fasting and with the drug interventions. Alanine levels were significantly decreased in all fasting conditions compared with the Fed condition (Fig. 2*G*), which is consistent with previous reports and indicates either an increased uptake by the liver for gluconeogenesis or a decreased release by skeletal muscle (47). Alanine levels were most reduced in Fast + Bort (down 66% compared with Fed, *P* < 0.0001) and significantly lower than Fast + CLQ (*P* < 0.01). Other glucogenic and ketogenic amino acids followed the same pattern, with lysine and valine being only significantly reduced in Fast + Bort (*P* < 0.05, Fig. 2, *H* and *I*).

The catabolism of nitrogen-containing substances like amino acids and purines causes the accumulation of toxic ammonia in the circulation (48). Since we found distinct levels of circulating amino acids and anticipated an effect of chloroquine and bortezomib on protein breakdown, we investigated levels of metabolites associated with the disposal of nitrogenous waste. The primary means to excrete ammonia in mammals is through conversion to urea in the liver. Urea cycle metabolite citrulline was most pronouncedly reduced in the Fast + Bort group (Fig. 2*J*). In addition, arginine levels were reduced in the Fast + Bort compared with the Fast and the Fast + CLQ groups (Fig. 2*K*). This finding is in line with our observation of maintained body and tissue mass with bortezomib during fasting, suggesting that the inhibition of proteasomal activity decreased protein breakdown and the accumulation of nitrogenous waste that needed to be processed through the urea cycle. Similarly, we found that uric acid levels were significantly lower in the Fast + Bort group compared with the Fast group (Fig. 2*L*). Uric acid is produced in response to the degradation of purines such as nucleic acids, indicating that the catabolism of these substances was impaired by bortezomib, too.

The disproportionate decrease in glucose with bortezomib indicates that the proteasome may either facilitate endogenous glucose production or limit systemic glucose utilization. The former is supported by the robust change in circulating ketone bodies and TCA metabolites.

### Molecular Signaling of Hepatic Anabolism Is Increased with Bortezomib but Not Chloroquine during Fasting

**Figure 3.**
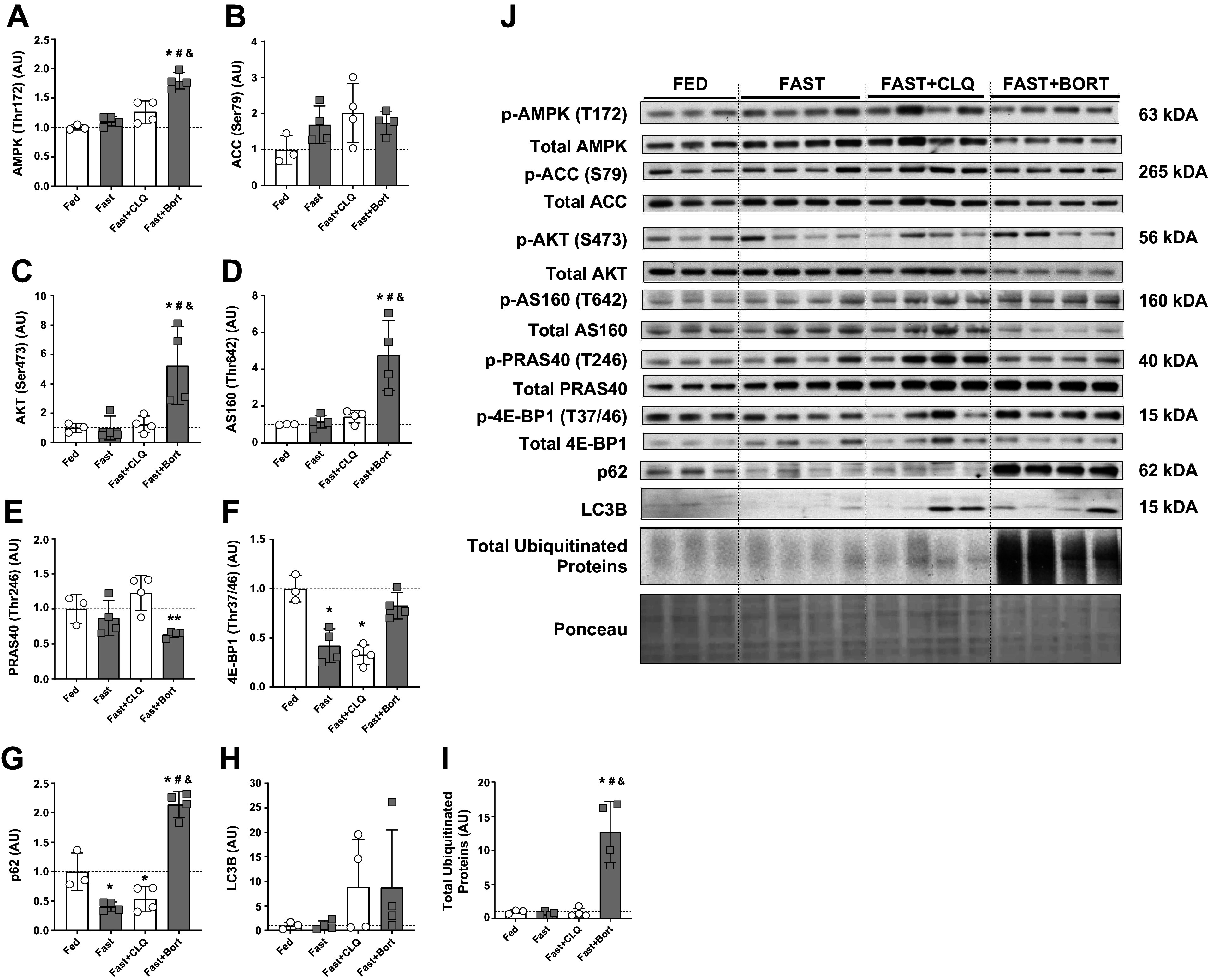
Molecular signaling of hepatic glycolysis and anabolism are increased with bortezomib but not chloroquine during fasting. Liver Western blot analysis. *A:* phospho-AMPK (T172) relative to total AMPK protein levels. *B:* phospho-ACC (S79) relative to total ACC protein levels. *C:* phospho-AKT (S473) relative to total AKT protein levels. *D:* phospho-AS160 (T642) relative to total ACC protein levels. *E:* phospho-PRAS40 (T246) relative to total PRAS40 protein levels. *F:* phospho-4E-BP1 (T37/46) relative to total 4E-BP1 protein levels. *G*: P62 protein levels normalized to total protein per lane (Ponceau). *H*: LC3B II protein levels normalized to total protein per lane (Ponceau). *I*: total ubiquitinated proteins per lane normalized to total protein per lane (Ponceau). *J*: representative blots. A one-way ANOVA and a Tukey post hoc multiple comparison test were used to assess group differences. Statistically significant differences to the Fed condition are denoted as * and ** (*P* < 0.05 and 0.01, respectively). Significant differences to the Fast group (*P* < 0.05) are denoted as #. Significant differences to the Fast+CLQ group (*P* < 0.05) are denoted as &. Bort, bortezomib; CLQ, chloroquine.

Next, we performed an assessment of molecular signaling pathways that regulate glucose, lipid, and protein metabolism in the liver to gain mechanistic insights into how the changes observed on the metabolic and macroscopic level are mediated by cell signaling. AMPK is a key regulator of glucose and lipid metabolism during fasting (11). We found that phospho-AMPK (Thr172/Total) was significantly higher in the Fast + Bort group compared with all other conditions (Fig. 3*A*), indicating that there was a more pronounced energy crisis with bortezomib than in the other fasting groups. Acetyl-CoA carboxylase (ACC) acts as a regulator of fatty acid metabolism by increasing fatty acid synthesis and suppressing fatty acid oxidation (49, 50); AMPK directly phosphorylates ACC at Ser79 (51). Phosphorylation of ACC varied widely in response to our fasting and drug interventions without any effect reaching significance (Fig. 3*B*). We used phospho-AKT (Ser473) as a readout of PI3K-mTOR activity in the liver. These levels were similar in all groups except for the Fast + Bort where there was an increase to 5.3-fold compared with Fed, driven by a significant reduction in total AKT protein (Fig. 3*C*). Recent reports have suggested that there could be a preservatory effect of AMPK toward AKT and cell survival in situations of severe stress (52, 53). AKT regulates glucose and protein metabolism, in part, by directly phosphorylating AKT-Substrate 160 (AS160) and PRAS40, respectively (54, 55). In line with our observation that AKT activity was increased in the Fast + Bort group but none of the other conditions, phospho-AS160 (T642) levels were increased to 4.8-fold in Fast + Bort compared with Fed and significantly higher than in the other conditions (Fig. 3*D*). Interestingly, phospho-PRAS40 (T246) levels tended to behave in opposite fashion with significantly lower activity in Fast + Bort compared with Fast + CLQ (Fig. 3*E*). Phosphorylated PRAS40 binds the scaffolding protein 14-3-3 and dissociates from Raptor, thereby allowing mTORC1 access to its downstream effector substrates (56). Once activated, mTORC1 phosphorylates 4E-BP1 to promote translation initiation and protein synthesis (57). We found that phospho-4E-BP1 (T37/46) levels were significantly decreased in the Fast and Fast + CLQ conditions, but not in the Fast + Bort condition compared with Fed (Fig. 3*F*). In addition, Fast + Bort levels were significantly higher than in the Fast or Fast + CLQ groups (Fig. 3*F*). Thus, our data support increased AKT activity toward AS160 but not PRAS40 with bortezomib, and a preservation of anabolic downstream signaling through 4E-BP1, indicating that the drug was able to partially restore hepatic PI3K/AKT/mTOR signaling during fasting.

Autophagy was assessed by measuring the abundance of p62 and LC3B II, both proteins known to be essential for this process. Interestingly, we found p62 to be significantly decreased in the Fast and the Fast + CLQ groups compared with the Fed condition, whereas levels in the Fast + Bort group were significantly higher compared with all other conditions (Fig. 3*G*). P62 is a cargo adaptor that is classically associated with autophagy, but recent work has shown that it interacts with ubiquitinated proteins to deliver them to the autophagosome or the proteasome (58), essentially acting as crossroads between the two processes (59). Our data provide strong support for these observations, showing a marked change in p62 with bortezomib during fasting (Fig. 3*G*). This suggests that either bortezomib has an inhibitory effect on autophagy or the increased presence of ubiquitinated proteins results in a compensatory elevation of p62 levels. Alternatively, p62 could be preferentially degraded by the ubiquitin-proteasome system and its blockage results in an accumulation of p62. LC3B II levels were on average 8.8-fold as high in the Fast + CLQ and the Fast + Bort groups compared with the Fed and Fast conditions but did not approach statistical significance due to large variation between mice in these groups (Fig. 3*H*). This could support the hypothesis that not only chloroquine but also bortezomib directly affected autophagy, albeit that LC3B II protein levels are notoriously difficult to interpret (60). Finally, we found that total ubiquitinated protein levels were 12.7-fold as high in the Fast + Bort group compared with Fed and significantly higher than in all other groups (*P* < 0.001; Fig. 3*I*). The accumulation of proteins tagged with ubiquitin offers a robust readout for the inhibition of protein degradation through the proteasome system with bortezomib, especially in the context of the known pharmacological effects of the drug (61).

### Systematically Inhibiting Autophagy and the Ubiquitin-Proteasome System Causes Molecular Signaling in Skeletal Muscle That Is Distinct from the Effects on the Liver

**Figure 4.**
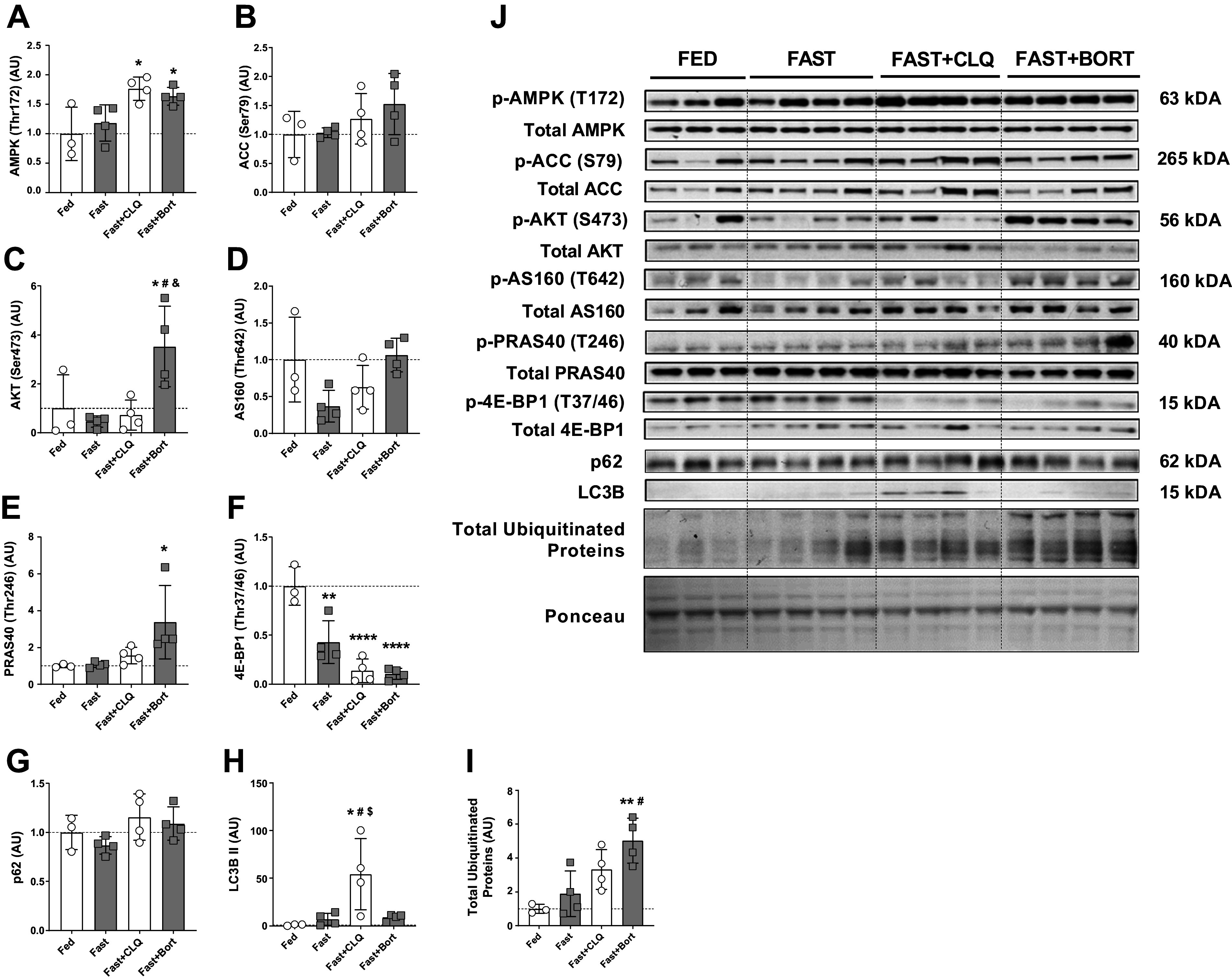
Systematically inhibiting autophagy and the ubiquitin-proteasome system causes molecular signaling in skeletal muscle that is distinct from the effects on the liver. Skeletal muscle (gastrocnemius) Western blot analysis. *A:* phospho-AMPK (T172) relative to total AMPK protein levels. *B*: phospho-ACC (S79) relative to total ACC protein levels. *C*: phospho-AKT (S473) relative to total AKT protein levels. *D*: phospho-AS160 (T642) relative to total ACC protein levels. *E*: phospho-PRAS40 (T246) relative to total PRAS40 protein levels. *F*: phospho-4E-BP1 (T37/46) relative to total 4E-BP1 protein levels. *G*: P62 protein levels normalized to total protein per lane (Ponceau). *H*: LC3B II protein levels normalized to total protein per lane (Ponceau). *I*: total ubiquitinated proteins per lane normalized to total protein per lane (Ponceau). *J*: representative blots. A one-way ANOVA and a Tukey post hoc multiple comparison test were used to assess group differences. Statistically significant differences to the Fed condition are denoted as *, **, and **** (*P* < 0.05, 0.01, or 0.0001, respectively). Significant differences to the Fast group (*P* < 0.05) are denoted as #. Significant differences to the Fast+CLQ group (*P* < 0.05) are denoted as &. Significant differences to the Fast+Bort group (*P* < 0.05) are denoted as $. Bort, bortezomib; CLQ, chloroquine.

Since skeletal muscle is the main deposit of amino acids in the body and to compare the molecular effects of our intervention across tissues, we investigated metabolic signaling in the gastrocnemius muscle of our mice. Phospho-AMPK (T172) was significantly increased in the Fast + CLQ and the Fast + Bort groups compared with Fed (Fig. 4*A*). Downstream AMPK target phospho-ACC (S79) was unchanged between the groups (Fig. 4*B*). Similar to what we observed in the liver (Fig. 3*C*), PI3K/mTOR/AKT signaling through phospho-AKT (S473) was 3.5-fold as high in the gastrocnemius of the Fast + Bort group compared with Fed and significantly higher than in all other conditions (Fig. 4*C*). Phospho-AS160 (T642) trended to be significantly decreased in the Fast compared with the Fed condition (*P* = 0.07) and trended to be higher in the Fast + Bort compared with the Fast condition (*P* = 0.06; Fig. 4*D*). Unlike in the liver (Fig. 3*E*), phosphorylation of AKT downstream target PRAS40 (T246) was significantly increased in the Fast + Bort group compared with the Fast condition (Fig. 4*E*). In addition, downstream mTORC1 target phospho-4E-BP1 (T37/46) was significantly decreased in all fasting conditions compared with Fed and trended to be lower in Fast + CLQ (*P* = 0.09) and Fast + BORT (*P* = 0.06) compared with Fast (Fig. 4*F*). This result indicates that fasting and our drug interventions affect liver and muscle differently, in keeping with the fact that macroscopic changes in tissue mass were more prevalent in the liver (Fig. 1*C*), whereas skeletal muscle mass was unchanged in all groups (Fig. 1*E*). This result is consistent with previous reports that showed skeletal muscle mass is preferentially preserved and degraded at a slower pace than other tissues like the liver and fat during nutrient deprivation (62).

Finally, we investigated signaling directly pertinent to the autophagy lysosome and the UPS in skeletal muscle. Unlike in the liver where we found a robust difference in p62 (Fig. 3*G*), protein levels of p62 in muscle were similar between the conditions (Fig. 4*G*). In contrast, we could only detect LC3B II in the Fast + CLQ group where protein levels were significantly higher than in all other conditions (Fig. 4*H*). This is consistent with other reports that showed an increase in LC3B II in skeletal muscle of mice that received a combined treatment of chloroquine and fasting (30). Similar to what we saw in the liver (Fig. 3*I*), total ubiquitinated protein levels were significantly increased in Fast + Bort compared with Fed and Fast (Fig. 4*I*). In line with other reports that showed chloroquine could affect ubiquitination in skeletal muscle (63), total ubiquitinated protein levels trended to be increased in Fast + CLQ compared with Fed (*P* = 0.06; Fig. 4*I*).

### Bortezomib Increases Global Muscle Protein Synthesis during Fasting

**Figure 5.**
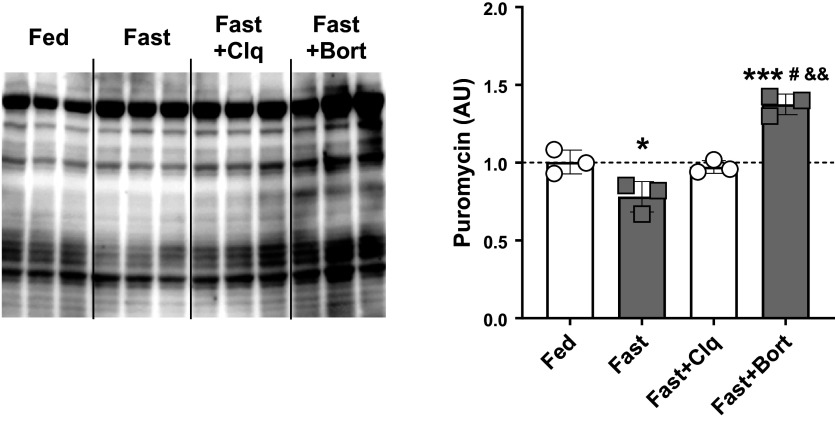
Global hepatic protein synthesis is reduced with overnight fasting but rescued through bortezomib treatment. Liver protein synthesis rates were assessed via the SUnSET method. Briefly, the tRNA analog puromycin was injected 30 min before tissue collection. Puromycin then was incorporated into every nascent peptide synthesized between injection and tissue collection. The presence of puromycin in the liver protein pool was then assessed via immunoblotting. Shown is the full membrane that was probed for puromycin (*left*) and the quantification of the total lane intensities (*right*). Fasting reduced hepatic protein synthesis rates compared with the Fed state, whereas Fast + CLQ remained unchanged. Bortezomib slightly increased protein synthesis rates compared with Fed and the other fasting groups. A one-way ANOVA and a Tukey post hoc multiple comparison test were used to assess group differences. Statistically significant differences to the Fed condition are denoted as * and *** (*P* < 0.05 or 0.001, respectively). Significant differences to the Fast group (*P* < 0.05) are denoted as #. Significant differences to the Fast+CLQ group (*P* < 0.01) are denoted as &&. CLQ, chloroquine; SUnSET, SUrface SEnsing of Translation.

Since tissue homeostasis and the preservation of mass are determined by the balance between degradation of molecules and biosynthesis, we assessed liver protein synthesis in our mice. We used the injection of puromycin, a tRNA analog known to be incorporated into any de novo peptide that is being synthesized after administration (64, 65). Through a puromycin antibody, in vivo global protein synthesis can then be assessed via immunoblot analysis as described previously (32, 66). We blotted puromycin levels in liver tissue from our experiment since the macroscopic changes in the liver seemed to closely mirror whole body changes in mass (Fig. 1, *B* and *C*). Interestingly, we found that fasting only slightly decreased protein synthesis whereas bortezomib in addition to fasting led to a modest but significant rise in hepatic protein synthesis (Fig. 5).

## DISCUSSION

This study investigated the contribution of autophagy and the UPS to changes in systemic and tissue metabolism in mice during fasting. We used well-characterized drugs to impair both processes and compared them with fed and fasted mice without drug treatment. Most importantly, we found that the selective 20S proteasome inhibitor, bortezomib, but not the autophagy inhibitor, chloroquine, maintained tissue and overall body weight in our mice during an 18 h overnight fast. Liver mass closely resembled the change in overall body weight, with significant decreases in the fasting and the fasting plus chloroquine groups but completely maintained tissue mass with fasting and bortezomib. We assessed serum metabolite levels through two independent methods and detected that the body weight maintaining effects of bortezomib were associated with exacerbated hypoglycemia and hyperketonemia.

In keeping with these findings, we observed a decrease in circulating glucose and increase in ketones associated with AMPK activation and preservation of insulin signaling through AKT and mTOR in the liver. Surprisingly, but in agreement with these observations, we also found a modest but significant increase in hepatic protein synthesis with bortezomib during fasting. This finding is in line with recent evidence for bortezomib to enhance protein synthesis in cell culture (67, 68). In addition, the observed effect of bortezomib on CaMKII in one of these studies (67) offers another possible explanation for how the drug affected AMPK activation in our study. Furthermore, the increase in hepatic protein synthesis in our study is consistent with the observed increase in AKT activity (Fig. 3, *C* and *D*) and the preservation of mTOR signaling through 4E-BP1 in the liver (Fig. 3*F*). This highlights the importance of AMPK and AKT signaling for cell survival in situations of energetic stress and supports other reports that found a positive relationship between AMPK, AKT, and anabolic signaling in those scenarios (52, 53, 69, 70).

We also found total ubiquitinated protein levels to be robustly increased in the liver with bortezomib but none of the other conditions, supporting that bortezomib did block UPS activity and the removal of ubiquitin-tagged proteins. Since proteostasis is determined by the balance between protein degradation and synthesis, together these data suggest that in our experiment, the positive effects of bortezomib on tissue mass were not just mediated through inhibition of proteolysis but also by elevating protein synthesis.

Although preserving tissue mass during acute starvation is a desirable outcome, prolonged, severe hypoglycemia can be fatal (71). Hypoglycemia is an effect that has been reported for bortezomib in clinical trials with cancer patients (72–74). Thus, the therapeutic applicability of bortezomib to ameliorate tissue loss in chronic scenarios is likely limited. Nevertheless, our finding that targeting the UPS was an effective strategy to offset the weight and tissue loss seen during fasting has implications for clinical challenges associated with energetic stress such as sarcopenia or cachexia (75, 76). Our observation that the UPS and not the autophagy-lysosome system was the primary mechanism of tissue degradation in our study is in line with other reports (77) and supports the finding that autophagy is indispensable for tissue preservation (23) and thus unlikely to be a suitable target for inhibition.

One of the limitations of our study is that we did not measure autophagic flux directly. However, the available methods to assess autophagic flux such as genetic models are imperfect and suffer from their own limitations (78). The phenotypical changes we have found in our study were robust, and we have investigated multiple tissues like serum, liver, and muscle, where we found coherent results that were supported by molecular signaling events and physiological data. Furthermore, we used validated drugs that are known to have a high specificity for inhibiting the 20S proteasome (61) and fusion of the autophagosome to the lysosome (21). In combination with positive controls of a fed and a fasted group without drug treatment and in the context of the available literature, we are confident that our results are reproducible.

Another potentially confounding factor is the possibility that our chloroquine dose was too low to successfully impair autophagy in our experiment. For example, there are reports that have previously failed to show a change in LC3B with a daily dose of 50 mg/kg body weight (79). However, this study did not combine chloroquine treatment with fasting. In another experiment that also used 50 mg/kg, the authors found an increase in LC3B that was further exacerbated by fasting (30). Therefore, it appears unlikely that the two doses of 50 mg/kg used in this study did not have the desired effect on autophagy. Indeed, we found significantly elevated total LC3B II levels in the gastrocnemius of CLQ-treated animals. Although still an imperfect way to assess autophagic flux, quantification of LC3B II in the presence of a known inhibitor of autophagosome-lysosome fusion such as chloroquine has been suggested as the most appropriate way to investigate the impairment of autophagy via immunoblotting (60).

Lastly, substrate utilization and fate can only be directly measured through tracer techniques such as stable isotopes (80). Looking at steady-state circulating metabolites alone leaves their origin and destination uncertain. Nevertheless, we provided context by not only measuring tissue weight, but also macromolecule composition (i.e., liver glycogen, protein, and TG), central signaling pathways, and physiological function (i.e., protein synthesis). In addition to the available information on overnight fasting, we believe that this has created the necessary framework to interpret our data in a meaningful manner.

In conclusion, blocking proteasomal activity circumvents fasting-induced tissue wasting in healthy mice during an 18 h overnight fast. Tissue maintenance was associated with altered systemic metabolism including decreased blood glucose and increased ketone bodies as well as preserved anabolic signaling and protein synthesis. Future research needs to directly trace the effect of bortezomib on substrate utilization to solidify our understanding of systemic, metabolic changes and to evaluate its safety for the treatment of tissue wasting during chronic energy crises. Additional experiments are also required to better delineate the interdependence of the proteasome and autophagy, which could help improve the specificity and efficiency of pharmaceutical interventions.

## DATA AVAILABILITY

Data will be made available upon reasonable request.

## GRANTS

This study was supported by institutional funds provided by Weill Cornell Medicine.

## DISCLOSURES

M.D.G. reports consulting or advisory roles with Scorpion Therapeutics; stock or other ownership interests in Faeth Therapeutics; honoraria from Novartis, Pfizer, Scorpion Therapeutics; patents, royalties, and other intellectual property with Weill Cornell Medicine. All outside of the scope of this manuscript.

## AUTHOR CONTRIBUTIONS

H.T.L., S.R.T., and M.D.G. conceived and designed research; H.T.L., S.R.T., M.A., T.P., T.A., and M.D.G. performed experiments; H.T.L., S.R.T., and M.D.G. analyzed data; H.T.L., S.R.T., and M.D.G. interpreted results of experiments; H.T.L. prepared figures; H.T.L. drafted manuscript; H.T.L., S.R.T., M.A., T.P., T.A., and M.D.G. edited and revised manuscript; H.T.L., S.R.T., M.A., T.P., T.A., and M.D.G. approved final version of manuscript.
